# An Overview of the Bodily Awareness Representation and Interoception: Insights and Progress in the Field of Neurorehabilitation Research

**DOI:** 10.3390/brainsci14040386

**Published:** 2024-04-16

**Authors:** Chiara Parma, Federica Doria, Aida Zulueta, Jacopo Lanzone, Marilisa Boscarino, Luca Giani, Christian Lunetta, Marta Vassallo, Eugenio Agostino Parati, Mario Picozzi, Davide Sattin

**Affiliations:** 1 Medicina Clinica e Sperimentale e Medical Humanities, PhD. Program, Insubria University, 21100 Varese, Italy; mvassallo2@uninsubria.it; 2 Istituti Clinici Scientifici Maugeri IRCCS, Health Directorate, Via Camaldoli 64, 20138 Milan, Italy; 62202191@mail.sfu.ac.at (F.D.); davide.sattin@icsmaugeri.it (D.S.); 3 Istituti Clinici Scientifici Maugeri IRCCS, Labion, Via Camaldoli 64, 20138 Milan, Italy; aida.zuluetamorales@icsmaugeri.it; 4 Neurorehabilitation Department, Istituti Clinici Scientifici Maugeri IRCCS, Via Camaldoli 64, 20138 Milan, Italy; jacopo.lanzone@icsmaugeri.it (J.L.); marilisa.boscarino@icsmaugeri.it (M.B.); luca.giani@icsmaugeri.it (L.G.); eugenio.parati@icsmaugeri.it (E.A.P.); 5 Amyotrophic Lateral Sclerosis Unit, Neurorehabilitation Department, Istituti Clinici Scientifici Maugeri IRCCS, Via Camaldoli 64, 20138 Milan, Italy; christian.lunetta@icsmaugeri.it; 6 Center for Clinical Ethics, Biotechnology and Life Sciences Department, Insubria University, 21100 Varese, Italy; mario.picozzi@uninsubria.it

**Keywords:** body awareness, interoception, rehabilitation, sensory inputs

## Abstract

In the last two decades, the scientific literature on so-called body representations has been increasing, and the notion of body awareness (BA) is particularly interesting for neurorehabilitation. In this article, we present results derived from recent studies on this representation, considering the different definitions and explicative models proposed as well as the empirical settings used to test it, providing an extensive overview of these issues. This article discusses the challenge of understanding how we integrate the sensory experiences of proprioception (knowing where our body is in space) and interoception (sensing internal bodily sensations, like hunger of thirst) with our perception of self. This is a difficult problem to analyze because our awareness of our body is inherently linked to our perspective, since the body is the means through which we interact with the world. Presenting the different viewpoints offered by recent theories on this concern, we highlighted that the neurorehabilitation and psychiatric settings offer two important fields useful for the study of BA because in them it is possible to analyze bodily representations by inducing/observing a controlled discrepancy between dysfunctional content and sensory inputs.

## 1. Introduction

The issues associated with the complex concept of bodily awareness are a puzzle in the cognitive sciences. This concept is based on the idea that we are consciously aware of the external world, its objects, our psychological state, as well as our feelings, thoughts, and perceptions and that this is something peculiar in humans. Accordingly, to this viewpoint, our awareness extends so much that our senses are able to point us toward our body, its presence, and its location. In this sense, the body is just like another object of the outside world. However, we are also able to perceive it from the inside, via several mechanisms such as proprioception, (i.e., the processing of the position and movement of limbs), balance, and interoception (defined by Charles Sherrington and before him by Hering-Breuer more than 100 years ago as the sensation presumably originated from the viscera [1]). Given these premises, it is clear that the awareness of our own bodies is able to generate a rich empirical and theoretical debate because of its coexistent subjective and objective nature.

But this is not the unique motive for which neuroscience focused its attention on the relationships among body awareness, proprioception, and interoception. Indeed, body awareness seems to be intrinsically relevant for the development and maintenance of one of the other mysteries of the living being: the “Sense of Self”, described as an integrative structure of the mind that organizes affective, cognitive, social, sensorimotor, and vegetative functions [2].

Considering the extensive literature and the widespread debate about the definition of the term “body awareness”, in this narrative review, we aim to provide an overview of body awareness (BA) and interoception by offering definitions and theoretical models. We will discuss the primary assessment approaches used to access these constructs, along with noteworthy empirical research that particularly focuses on the rehabilitation of neurological disorders associated with awareness deficits. Our objective is to highlight the complex role and interaction between BA and interoception, along with the role of other constructs such as proprioception and exteroception, in shaping and maintaining bodily representations, and, ultimately, contributing to the “Self”.

## 2. History and Definition of the Term Body Awareness

The contemporary debate on this issue started with Merleau-Ponty [3] in 1945, and since then, many others have proposed solutions and interpretations of this issue.

Merlau-Ponty argues that our relationship with our bodies goes beyond mere possession but we “inhabit” and live with them every moment of our life. Consequently, he distinguished between two notions of the body: the “objective body”, which is like any other physical object and has quantifiable attributes like size and weight. This is the body we assess when we employ a tape measure to determine our weight. Secondly, on the other hand, he introduces the concept of the “lived body” referring to the body through which we touch and feel and move. And this latter notion, he wrote, grounds us as being “body-subjects” before all else. Merleau-Ponty drew attention to the body not as merely a lump of matter, but as the breathing, beating center of our experience—the “lived” body, in contrast to the theoretical position of perfect objectivity. His innovation focused on how we always perceive things from a particular perspective, how the particular configuration of your body means that you never directly see the back of your neck. Through perception, the body is always called upon to engage, to choose, to focus on the world before any verbal reflection takes place, setting the scene for whatever we go on to reflectively think, say, and do. This is why Merleau-Ponty concluded that bodily engagement with the world is more basic than deliberation about it: not as a way of privileging the physical over the mental, but as a description of what it is like to move through the world, mind and body working as one.

Merleau-Ponty offers the following example: “When I press my two hands against one another … [I encounter] an ambiguous organization in which the two hands can alternate in the function of ‘touching’ and ‘touched’” [3]. Which hand is touching and which is touched? This ambiguity extends to our interactions with other people. Ultimately, Merleau-Ponty believed that bodily awareness of this “intertwining” fosters our sensitivity towards others, what Mazis calls “embodiment’s access to the heart”. Intimacy, connection, and compassion rest on our perception of one another, less as intellectual grasp of the other as a “conscious agent”, but more as a felt sense of this embodied, sensitive, and vulnerable being before me.

However, bodily awareness still represents an unsolved and controversial topic in several fields, and a definitive and proven interpretative theory has not been proposed yet [4].

This is probably because the more we know about human physiology and psychology, the more difficult becomes to pinpoint this particular phenomenon to some specific and convincing explanation, especially because we are still debating about the nature of consciousness and whether it is related to bodily awareness [5,6].

At a sensorial level, we receive a constant flow of information, both about our own body and the outside world; such information can be interpreted as being external, internal or both, but in each case this never-ending flow passes through us. Furthermore, we can actually see and interact with our own body, but we also are equipped with several receptors that are able to convey the information we receive about our own body, such as: the position of our extremities, our balance at a certain time and sometimes even our physiological state, e.g., when we feel pain, we are receiving information about our own physiological state [7] as we reported in the above paragraph.

Body awareness has been described in numerous ways but, in the last decades, a multidimensional conceptualization was provided by Mehling et al. [8] (p. 1), who defined BA as: “the subjective, phenomenological aspect of proprioception and interoception that enters conscious awareness, which is modifiable by mental processes including attention, interpretation, appraisal, beliefs, memories, conditioning, attitudes and affect”, highlighting in this definition the intrinsic interactive process that involves ourselves as “the awareness of the body’s physiological states, processes (including pain and emotion) and actions (including movement), and is shaped by an individual’s attitudes, perceptions, beliefs and social/cultural context experiences” [9] (p. 2).

Recently, researchers studying BA have tried to explain better the relationship between BA and proprioception and interoception already in their definition of BA, as it is possible to note in the definition proposed by Mehling that as: “awareness of sensations coming from inside the body, mainly related to proprioception and interoception, even if it interacts with exteroceptive stimuli and thoughts, excluding exteroceptive channels altogether” or, as proposed more recently by Ainley and Tsakiris [10] (p. 4) the “Body awareness is the perception of bodily states, processes and actions that is presumed to originate from sensory proprioceptive and interoceptive afferents and that an individual has the capacity to be aware of”.

As an attentive reader may notice, all the above-reported definitions consider BA a multidimensional concept requiring deep attention and careful examination of the components considered intrinsic to the concept itself. This is the reason why in the next sessions of this work we will try to summarize the most recent perspectives related to interoception, to discuss BA and, at the same time, what is the evidence related to interoception in parallel.

## 3. History and Definition of Interoception

In the early stages, interoception was conceptualized as the processing of signals coming from the viscera initially believed to be solely from the intestine [11]. Subsequently, the definition has encompassed additional systems such as the cardiovascular, gastrointestinal, respiratory, and genitourinary systems, distinguishing itself from proprioception and exteroception (for definitions see Table 1).

Interoception has garnered renewed attention in the last two decades and it has been recently described as the set of “processes by which an organism senses, interprets, integrates, and regulates signals originating from within itself” [12] (p. 4) or, in a more shared definition by Khalsa and collaborators [13] (p. 501) as it “refers collectively to the processing of internal bodily stimuli by the nervous system” and “broadly relates to all physiological tissues that relay a signal to the central nervous system about the current state of the body, including the skin and skeletal/smooth muscle fibers, via lamina I spinothalamic afferents”.

Analyzing some studies related to interoception, it was found that this issue is conceptualized as composed of different parts that we can associate with the BA concept in different perspectives. For example, some theories consider interoception as composed by proprioception and exteroception, grouped together under the name “somatic sense”, which refers to the sense of signals that come from the muscles, joints, connective tissues, and skin [14], in particular to distinguish them from homeostatic pathways. Homeostatic pathways, termed the “interoceptive system”, comprise specific neurons in the spinal cord and the solitary nucleus (NTS), sensory fibers (Aδ and C small-diameter sensory fibers), lower brain regions (such as the thalamus), and higher brain regions (including the dorsal posterior insula and the anterior insular cortex), which enable the central nervous system (CNS) to monitor the entire body’s physiological state and respond to maintain internal stability (homeostasis) [14,15]. The non-homeostatic system instead is composed of large-diameter sensory fibers of Aβ cells from the skin and from muscle and joints. This juxtaposition between homeostatic pathways (interoception) and non-homeostatic pathways (or somatic system) (for a deeper description see Desmedt et al., 2023 [16]) comes from the distinction between visceral and somatosensory sensations, which arises from differences in the neural pathways involved in processing these signals [15], i.e., primary and secondary somatosensory cortices for somatic sensations, whereas visceral signals are transmitted from to brainstem to the thalamus and then to the insular cortex.

This distinction also generates different subjective experiences important for body awareness processing: visceral sensations tend to be more widespread, whereas somatosensory sensations are more localized. Pain and temperature processing are categorized as somatic signals and are mediated by the somatosensory cortices, excluding them from the definition of interoception, which focuses on internal bodily sensations contributing to one’s sense of self and well-being.

Currently, other researchers adopt a narrow interpretation of interoception, confining it to visceroception [17,18], but the wide consensus leans towards Craig’s (2009) [19] broader approach [20,21]. He defined interoception as involving both conscious and unconscious processing of a wide range of signals through homeostatic pathways (mechanical, thermal, or chemical conditions of tissues, including skin) that provide information about physiological needs, organ integrity, and homeostatic control.

This definition of interoception involves major, visceral systems such as the gastrointestinal, respiratory, and cardiovascular systems, and also cutaneous sensations according to some models that expand the definition of interoception to accommodate other motivationally important physiological signals. Then, interoception contributes to the core aspect of bodily awareness, defined as attentional focus on and awareness of internal body sensations [22] (Mehling et al., 2009). However, in an interesting work of Desmedt and colleagues [16] some limitations were highlighted regarding the recent definitions of interoception (e.g., lack of clarity regarding the involvement of conscious or unconscious perceptions, the brain regions and physiological systems engagement, debate about the inclusion of some aspects of exteroception in the definition of interoception etc.), proposing a new definition of interoception in trying to overcome these issues: “interoception includes the top down and bottom-up processed by which an organism senses, interprets, and integrates signals from within itself and below the skin, across conscious and nonconscious levels” (p. 12). In consequence, they consider the skin as the boundary of interoception, and they excluded from its definition the signals coming from temperature and pain through the external surface of the skin.

In contemporary interoception research, two main conceptualizations of conscious interoception dimensions have been widely shared to standardized terminology regarding its components, considering that it varies a lot across the field. The first one was provided by Garfinkel and colleagues [23] with their three-dimension model based on measurement types. With the aim to extend the terminology, Khalsa et al. [13] proposed a model with eight dimensions, where they have replaced the term “interoceptive awareness” with “interoceptive insight”. In Table 1, we provide the definitions of the different components of interoception according to Garfinkel et al. [23], Khalsa et al. [13], and Pollatos et al. [24]; the latter have added a fourth component on the Garfinkel’s model (emotional evaluation of interoceptive signals).

**Table 1:** Different dimensions of interoception according to Garfinkel et al. [23], Khalsa et al. [13], Heim et al. [25], and Pollatos and Herbert [24], who proposed a fourth component (IE). We also differentiate interoception from exteroception and proprioception. Instead, body awareness is constructed by signals originating both from within and outside the body; therefore, we consider it to be an umbrella term that encompasses the previous ones.

Definitions			
Garfinkel’s Definitions		Khalsa’s Definitions	
Interoceptive accuracy (Iacc)	Objective accuracy in detecting signals from within the body such as heart beating, hunger or thirst. E.g., Accurately detecting heart rate	The presence or absence of report.	Detection
Interoceptive sensibility (IS)	Self-perceive dispositional tendency and ability to focus on body states such as muscle tension, hunger, dry mouth, and the capacity to detect them. E.g., Noticing changes in heart rate	Reporting subjective experiences and judging their outcomes.	Self-report
Interoceptive awareness (IA), metacognition	Metacognitive awareness of interoceptive accuracy. It is the correspondence between the objective performance during an IAcc task and the self-reported confidence in this performance. E.g., Accurately guessing one’s own level of IAcc		Interoceptive insight
		The perceived intensity of internal states.	Magnitude
		Localize sensations and differentiate them from other non-interoceptive sensations.	Discrimination
		Capacity to focus attentional resources on internal states.	Attention
		Correct and precise monitoring of internal signals.	Accuracy
		The self-perceived tendency to focus on internal states.	Sensibility
Emotional evaluation of interoceptive signals (IE)	Interpretation of any bodily sensation occurring or being paid attention to in a certain setting.		
Exteroception	Perception of external environment. The processing of signals coming from the outside, located on the external surface of the body, in other words the skin.		
Proprioception	Reflects the position of the body in the space. The processing of limb position and movements.		

Interoception has a significant role in a variety of fundamental psychological functions; for example, it provides the basis for the self and self-awareness and has a significant contribution to decision making [26,27], emotional regulation, and reward seeking [28,29]. In the next sections, we reported results of studies that are important for the discussion of the relationship between interoception and BA for our point of view.

## 4. Explicative Models of Body Awareness and Interoception

In comparison with the modern definition of interoception, the concept of body awareness was developed earlier. BA represents a more integrative approach to subjective body experience, based on the integration of several interoceptive sensory channels, as well as exteroceptive modalities. BA has also been described as an umbrella term that encompasses interoceptive and somatosensory awareness [19,22,30]. The two constructs of BA and interoception show considerable overlaps, in particular BA with the dimensions of interoception sensibility and interoceptive awareness, as it emphasizes the involvement of perceptual processes and conscious awareness.

The construct of BA can be explained well by the modern evolutionary approach to interoception, which stresses the adaptive importance of multimodal integration of body-related information in the mid-insula [15]. This integrated feeling of the body plays a role in homeostatic regulation, the generation of the feeling of being alive, and also in the emergence of emotional feelings. However, it remains unclear how individual interoceptive channels may contribute to BA and how they modify it.

Regarding interoception, an interesting approach was derived from neuroscientific research. “Predictive coding models” propose that the brain actively generates explanations for the stimuli it encounters. This means that all sensory perceptions, whether incoming from the external world or from interoceptive signals, are constructed by the brain, postulating that the core function of the brain is to minimize prediction errors with respect to a generative model of the world [31]. It is possible through a mechanism that compares the brain’s anticipation or prediction of sensations with the incoming sensations in an active and iterative process. These models suggest that minimizing “prediction errors” (PE) is adaptive for an organism’s survival. Prediction errors refer to the differences between the sensory sensations and the sensory inputs predicted by the internal models of the world and the body. When focusing on interoception, all sensory experiences reflect predictions about the body and the world. Specific “interoceptive predictive coding models”, such as the one proposed by Seth and Friston [32], propose that interoceptive experience reflects predictions about the expected state of the body, which are constrained by ascending visceral sensations. Homeostasis occurs when the PE is small or non-existent, whereas lower interoceptive accuracy (IAcc) reflects more “noisy” and less reliable and precise interoceptive information [33]. In this context, clinical symptoms of psychiatric and psychosomatic disorders related to IAcc dysfunction may be viewed as reflecting “dysbalances” in minimizing PEs within the interoceptive system [20]. Eating disorders, for example, are characterized by a significant impairment of the “sense of self” [2], with dysfunctional interoception at its core. They exhibit a greater malleability of the bodily self and tend to consider the body as an object, indicating a greater self-objectification tendency, which is an overfocus and reliance on exteroceptive stimuli of the body (i.e., vision). In fact, it has been discovered that individuals with eating disorders and impaired IAcc present more intense body-illusion effects in the rubber hand illusion paradigm [34] and body image representation seems to be a particular feature in these subjects (for an extensive review on this topic, see also Sattin et al. [35]). Interestingly, the definition proposed by Desmedt and colleagues [16], reported above, states that interoception involves both bottom-up and top-down processes, and that is completely consistent with the active inference theory of Seth and Friston [32] and with others’ recent computational models (e.g., [36,37]). This indicates that perceptual outcomes are often influenced by both types of processes. For example, individuals are more likely to feel abdominal pain if they anticipate it (e.g., after they have overeaten) and there is an actual painful stimulus (e.g., their stomach is full).

The studies supporting the predictive models approaches are increasing and—such as, for example, the model of Friston, based on the free-energy principle and Bayesian inference [38]—have also been applied in BA research, for example in the investigation of anosognosia for hemiplegia [39,40] and self-recognition [41]. This framework represents an alternative perspective to the modular view of neural systems, motor functions, and self-awareness. Furthermore, it has the potential to provide a comprehensive understanding of the clinical dissociations observed in BA deficits, which will be discussed in detail later.

It is difficult to critically discuss all the models presented considering the multivariable concerns and the complex system they try to explain. In particular, if we analyze some sentences reported above found in the cited articles, we find terms like “balance/disbalance” or “direct/indirect” effect but, in our perspective, the presence of causal intermediaries does not necessarily entail that perception is experientially and/or epistemically indirect. Reporting an example by Hopp [42] (p. 163), the fact that our perceptual access to spatio-temporal objects in the world is enabled and underpinned by various sub-personal mechanisms and non-conscious cognitive processes does not necessarily entail that we therefore fail to see the objects as they are in themselves. Rather, one might view the cognitive processing as that which makes it possible for us to experience those objects in the first place (see also Clark and Zahavi for a critical analysis on this issue [43,44]). In other words, an interoceptive deficit does not necessarily mean a body awareness deficit, as a distorted body representation can occur without interoceptive impairment in some conditions.

Finally, to conclude the discussion about the possible explicative top-down models of BA and interoception in an alternative perspective, an interesting study useful for interpretation of interactions among body awareness and interoception concerns another species: the fruit fly Drosophila [45]. In this study, fruit flies were shown as able to perceive the size of their own body by acquiring such information from visual feedback during deambulation, because of a neural cluster responsible for the maintenance of the body size memory. During this experiment, the authors successfully observed the flies acquiring body size memory through visual experience of stripes during a walking task. As a matter of fact, such insects when challenged with an obstacle in their way tended to evaluate predictively whether and how such obstacle could have been surpassed and whether the actual gap they had to pass through was of an adequate size or not; in the latter case, the majority of them would not even attempt to cross, i.e., the fruit flies successfully predicted their body size through the visual evaluation of their surrounding space. In this particular study, it has been postulated [46] that such bodily awareness showed by the flies had nothing to do with their biology, but it was linked to a special neural cluster of the central complex neurons. Such neurons are specifically the Δ7 neurons and they seem to be responsible for BA in the fruit fly Drosophila. However, although the authors wrote about BA in the title of their article, the relationship between body metrics, awareness, and memory is still controversial and far from being clear, as described above.

Interestingly, nevertheless, it is plausible [47] that, similarly to flies, humans have a much larger neural cluster or even a neural network made up of Δ7 neurons, which could be responsible for our own bodily awareness, but this has not been proven yet. Either way, if the physiological explanation of our bodily awareness resides in our brain connectivity, this needs to be proven as soon as possible, especially for clinical reasons [48].

In conclusion, the theoretical frameworks described could offer interesting input for future models able to explain BA and its interaction with interoception, but the work to carry out is huge yet. However, to foster the way for the development of interpretative models also considering the bottom-up approach (the rehabilitative perspective nature of this work), in the next section we focus our attention on some empirical results derived from studies on BA and interoceptive analysis in healthy subjects or patients with neurological disorders.

## 5. Empirical Research and Neural Substrates of Body Awareness and Interoception

Empirical research on bodily awareness has not always been conclusive, and many theories have been proposed, but none can be considered final at the moment. Not long ago [49], it was postulated that the insular cortex is the part of our brains where bodily awareness has its seat. Based on neuroanatomical data, the insula is a significant region of the brain that contributes to various aspects of human experience by integrating a variety of sensory signals originating from the body and combining them with emotional and motivational factors. It plays different roles, including its involvement in interoception, emotional processing, and self-regulation. In fact, it is part of the “interoceptive neural network” that also includes somatosensory and somatomotor cortices, anterior cingulate cortex, and prefrontal cortices, in particular the ventromedial prefrontal cortex and the dorsolateral prefrontal cortex [50]. This network is important for monitoring the internal emotional and viscerosensory state for emotional processing and reactivity and for the self-regulation of feelings and behavior [51]. Furthermore, different portions of the insula seem to be engaged in different and successive steps of neural processing: raw interoceptive signals from visceral changes and pain first project to the posterior insula and integrate with motivational and hedonic information as they progress to the anterior insula.

In order to observe the typical insular behavior, one should consider data from pathological settings; in fact, for example, an insular involvement in anxiety disorders and addiction has been observed. Research suggests a connection between insular activity and BA [52]: people who are highly aware of their bodily states may exhibit increased interoceptive prediction [53] (i.e., they might be increasingly able to predict future illness or altered physical states), which could predispose them to anxiety disorders, particularly in those with post-traumatic stress disorder (PTSD). Studies [54] have found correlations between accuracy in perceiving one’s own heartbeat and activity in the right anterior insular cortex, suggesting a potential link between insular function and anxiety disorders. Moreover, it could be possible that a variation in the insular activity may lead to an increased accuracy in the prediction of future events, and this could be a risk factor for developing an anxiety disorder.

The insula also plays a central role in addiction; in fact, fMRI studies [55] on humans have demonstrated insular activation during drug craving and drug consumption. Additionally, lesions in the insula have been associated with disruptions in addictive behaviors, such as smoking habits in patients with nicotine addiction [56].

However, a direct correlation between the insula and BA has yet to be empirically established, suggesting that it is more likely that BA may depend on broader neural connectivity and functional networks. The examination of patients with brain injury who show specific impairments in awareness can enhance our understanding of this matter. Anosognosia for hemiplegia (AHP), as a prototypical disorder of BA, is a condition that arises from right brain damage, where patients deny the presence of their contralesional motor deficits [57]. Motor anosognosia can affect specific limbs (and selectively upper or lower limbs) [58] and can be modality-specific (it can involve either motor or sensory impairment; this last case is called anosognosia for hemianesthesia) [59]. It has been shown that this condition is caused by lesions in specific regions, such as the ones of motor planning and motor control functions, like the premotor cortex [60] or posterior insula [61]. However, more recent studies [62,63] have revealed a more complex network of areas involved in awareness, suggesting that AHP is a disorder with multiple components caused by lesions in complex and widespread cortical–subcortical anatomical networks, rather than isolated regions [39]. These regions include the Rolandic operculum, the insula, the superior temporal gyri, as well as subcortically basal ganglia and white matter, particularly the superior corona radiate, arcuate fasciculus, and the part of the ventral, superior longitudinal fasciculus [63]. In this last study, it was specifically highlighted that subcortical structures and white matter tracts may be crucial for supporting the fundamentals for body ownership and control over body parts. Nevertheless, a precise and current understanding of our motor abilities may also rely on intact functioning in cortical regions, which facilitate higher-level interpretations of the body’s present state.

Other neurological conditions of our interest include anosognosia for hemianesthesia (AHA) and somatoparaphrenia (SP); the latter presents an alteration of the sense of ownership (towards parts of our body), which is a crucial aspect of bodily representation. SP is a neuropsychological condition where individuals have a delusional belief that their limbs belong to someone else. On the other hand, AHA is characterized by preserved ownership of one’s limbs but reduced awareness of sensory deficits; usually, these patients deny somatosensory deficits even when they are evident upon clinical examination. AHA is typically seen in patients with right hemisphere lesions and is considered a productive symptom of distorted bodily representation [64]. Research [65] has shown that patients with SP exhibit reduced responses to sensory threats approaching the body, indicating a significant detachment of the affected body part from the patient’s bodily representation. This phenomenon is specific to SP and not observed in AHA, suggesting that body awareness deficits in these two conditions occur at different levels, despite both being associated with right hemisphere lesions and often co-occurring. SP may not only disrupt bodily representation but also affect the interaction between the body and space, leading to reduced monitoring of a specific region of peripersonal space for potential threats. These findings highlight the complexity of body representation and its susceptibility to various levels of impairment following brain damage.

Moreover, the literature in recent years has proposed that BA may develop from the body schema and body image considered as representations of our body—the former unconscious and related to the surrounding space, the latter conscious [66]. Consequently, the neural substrates supporting the body schema and body image could potentially be some of the neural substrates of BA [35,67].

## 6. Assessment of Body Awareness and Interoception

Previous research has uncovered interesting distinctions between implicit and explicit awareness. This discrepancy was particularly observed in patients with AHP that can exhibit different levels of awareness simultaneously. These patients may have implicit knowledge of their impairments, but they tend to explicitly deny them [58,62]. To assess explicit awareness, verbal declarations regarding movement abilities are directly evaluated, whereas implicit awareness is deduced from behavioral observations. This dissociation between explicit and implicit awareness has been explored using verbal [62] or motor paradigms [58], revealing partially convergent anatomical patterns. Research on AHP can be crucial for enhancing treatment approaches and gaining understanding of the neurocognitive mechanisms underlying motor awareness. However, many studies rely on assessments with acknowledged limitations. In a notable study [68], a new tool called the Motor Unawareness Assessment (MUNA) was tested to create a psychometrically validated measure of AHP. Five key factors were found: explicit motor awareness, implicit motor awareness, impaired sense of ownership, agency and illusory movement, and emotional reactions. These factors offer a detailed profile of participants’ awareness levels and help differentiate various aspects of AHP.

In another study, a systematic review by Mehling et al. [22] different articles on BA were compared with the aim to find robust questionnaires useful for rehabilitation. In the review, Mehling and colleagues [22] saw that many self-report instruments of body awareness are developed only to measure anxiety-related symptoms or body image, so they compared many self-report instruments in order to better understand which domain of the construct they measured. They found 39 instruments related to body awareness, between which they selected 12 instruments that satisfied psychometric standards. These are the Body Intelligence Scale (BIS) [69], the Body Responsiveness Questionnaire (BRQ) [70], the Body Awareness Measure (BAM) [71], the Timer Questionnaire (TQ) [72], the Scale of Body Awareness (SBA) [73], the Questionario di Consapevolezza Corporea (QCC) [74], the Private Body Consciousness Sub-Scale (PBCS) of the Body Consciousness Questionnaire (BCQ) [75], the “Awareness”, “Stress Response”, and “Autonomic Nervous System Reactivity” subscales of the Body Perception Questionnaire (BPQ) [76], the Scale of Body Connection (SBC) [77], the Body Vigilance Scale (BVS) [78], the Body Awareness Questionnaire (BAQ) [79], and the Health Consciousness (HC) subscale of the Multidimensional Health Questionnaire (MHQ) [80] (see Mehling et al. [22], for a detail description of the questionnaires). Table 2 lists the items constituting the BAQ [79] and the BPQ [76].

They singled out as solid psychometric instruments only the BAQ and the PBCS, followed by the BVS and SBC. The BAQ is unidimensional and explicitly excludes attention to pain sensations and emotions, leaving out important information about body awareness as a conscious focus on physical sensations such as pain or sensory aspects of emotions. Therefore, all these instruments lack a multidimensional view of the construct.

Also, none of these tools were validated against an objective measurement. Heart rate detection accuracy [81,82] is the most common objective measure used to assess body awareness in interoception research. Anyway, it focuses only on a single element of interoception, the heart, which has not been reviewed in any awareness training approaches but has the advantage to be less susceptible to voluntary manipulation compared to respiration. Different mind–body approaches, like meditation or other forms of body awareness enhancement, focus on training awareness of breathing rather than heartbeat. It is still uncertain which aspects of BA these techniques increased, although the scientific literature in this area has been increased in the last years.

Two other instruments commonly used to assess BA and interoception are the Functional Body Sensation Questionnaire (FBSQ) [83] and the Multidimensional Assessment of Interoception (MAIA) [9]. These two questionnaires measure different aspects of BA; the first focuses on perception, differentiation, and body sensations during emotional experiences, and the MAIA evaluates attention, attitudes, and reactions towards emotional- and non-emotional-related body sensations. The FBSQ investigates three dimensions, namely Perception, Differentiation, and Emotion regulation. The sub-scale which measures differentiation of emotion-related sensations is not included in the MAIA. The MAIA questionnaire specifically aims to measure the interoceptive sensibility (IS) and has eight sub-dimensions, which are “self-regulation”, “noticing”, “not-distracting”, “not-worrying”, “attention regulation”, “emotional awareness”, “body listening”, and “trusting”. Some items of the MAIA assessing IS are expressed in a way that highlights hypersensitivity to bodily sensations, while others are formulated in a more neutral way. Another interesting questionnaire for BA evaluation is the Body Perception Disturbance (BPD) [84] which was developed in pain research to measure different parameters both on upper and lower limbs. These are the physical awareness of limb ownership and position, attention required to attend to limb, emotional feeling toward limb, difference in size, temperature, pressure, weight, and the description/mental image of body parts. A study conducted by Serrada and colleagues [67] revealed that BPD appears to be a more suitable tool for assessing BA in stroke patients when compared to MAIA. This is due to its incorporation of relevant components of BA, including structural features and body schema information. In Table 2 and Table 3, we report the principal measures used to assess body awareness and interoceptive components, respectively, and their therapeutic applications.

## 7. Rehabilitation Approaches

In neurological rehabilitation practice, both body awareness and interoception play significant roles, yet they are distinct constructs that can be investigated and utilized in different ways. It has been seen that many disorders have problems in BA such as chronic pain, anxiety, depression, obesity, PTSD, etc. Also, different neurological conditions, such as stroke or traumatic brain injury (TBI), could be associated with BA impairment. In neurological rehabilitation practice, body awareness can be explored and enhanced through various techniques, such as body and sensory awareness exercises and activities that promote movement awareness. The goal is to improve body perception and control, reduce the risk of falls, enhance motor functionality, and promote a better quality of life. Interoception can, instead, be investigated and utilized to better understand how patients perceive and respond to internal signals from their bodies, which may be influenced by neurological conditions such as stroke, TBI, multiple sclerosis, and other disorders. The goal is often to enhance awareness and management of these internal sensations to improve physiological regulation and response to therapeutic interventions.

### 7.1. Mind-Body Approaches—Enhancement of Embodied Self

Currently, there are many therapeutic approaches aiming to enhance BA classified as mind–body approaches, which include yoga [70,92], TaiChi, massage [93,94], Body-Oriented Psychotherapy [95], mindfulness-based therapies/meditation [96], Feldenkrais [97], Alexander Method [98], and Breath Therapy [99]. Other related therapeutic approaches are the Body Awareness Therapy (BAT) or Body Awareness Program (BAP) [100,101], which aim to develop a non-judgmental “mindfulness”, “a quality of relating to one’s experience with an orientation of curiosity, experiential openness, and acceptance” [102].

A study of Pérez-Peña and colleagues [103] investigated the role of mindfulness-based interventions (MBIs) on indirect measures of BA and self-report questionnaires on healthy subjects recruited on a voluntary basis. The questionnaires related to BA were the FBSQ [83] and the MAIA [9], described above, used to study BA conceived as a mindful and open attention to body sensations and how they can be used for self-regulation.

Self-report questionnaires have the problem to assess some aspects of BA that participants may not be aware of [9]; in the study, in addition an indirect measure of BA, the Modified Autobiographical Memory Test (mAMT) [104] was used, which consists of a memory task that involves counting the number of spontaneous references to body sensations, allowing an evaluation of the attention allocated to body sensations. It is an indirect measure because it does not explicitly ask the participant to collect the body sensations, but it records the spontaneous tendency to report them while emotional personal events are also collected. In the study, the AMT was modified by adding another task called the Autobiographical Memory Description Task. In the task, participants are asked to recall 6 out of 10 keywords that pertain to a remembered event and then are asked to provide as much detail as possible about their state during the event constituting the recall. These details regarding bodily sensations are referred to as the number of spontaneous references to bodily sensations and index of attention focused on the body when describing an emotional experience.

In the study, the authors examined, in particular, the mediating role of BA in the relationship between MBI and symptomatology in order to understand the role of MBI on cognitive processes like experiential avoidance, rumination, self-efficacy, and self-discrepancy (which was found as mediating factors in explaining MB practices) [105,106]. They record self-reported and indirect measures of BA during an 8-week Mindfulness-Based Cognitive Therapy program (MBCT), which teaches a non-judgmental, curious, and accepting attitude toward one’s experiences. Giving that BA acts in conjunction with other psychological processes, such as decentering [107], multiple mediator models were used in the study, with BA as the first mediator and other psychological variables as second mediators (i.e., reduction in unconstructive rumination and in experiential avoidance, reduction of the actual–ideal self-discrepancy gap, improvement of emotional regulation self-efficacy). This agreed with a study of Fissler and colleagues [107] that investigated the effect of MBCT on depressive symptoms, consisting of the development of functional strategies regarding regulatory and belief-related aspects of BA and of a fine ability to decenter from unhelpful beliefs or mindsets.

In the study of Pérez-Peña and colleagues [103], there was a significant reduction in psychological symptomatology after MBI, and this effect was mediated by some regulatory and belief-related dimensions of self-reported BA (i.e., attention regulation, self-regulation, body listening, and trusting), in particular from the MAIA items. Interestingly, they found an effect of MBI on the self-reported BA but not on the indirect measure of BA, and many correlations between BA questionnaires and mAMT were small, which may be caused by a lack of sensitivity or by the fact that the measures assess different constructs or different aspects of BA. These results are in line with previous research where self-report measures of BA do not correlate with other types of measures like behavioral measures [23,108]; for example, the heartbeat counting task was widely criticized because it relies on non-interoceptive processes [109]. It is necessary to validate all these kinds of measures of BA and to clarify which specific aspects of BA they access. In fact, one advantage of Perez-Pena’s study is that it employs a combination of techniques that measure both explicit and implicit aspects of BA. However, the questionnaires used lack sensitivity as they measure different aspects of BA, resulting in little consistency in measurement. This is an aspect to consider when analyzing these data.

MBIs may thus improve adaptive BA by teaching people to pay attention to body parts at rest (e.g., body scan and breathing meditation) and in movement (e.g., yoga and mindful walking) in a curious, open, and accepting way [110,111]. The literature has provided evidence that mindfulness practices can improve interoception [107,112] and proprioception. For example, it is associated with better motor performance and balance, slower and better accurate body movements, and higher awareness of perceptual–motor conflict in a visuo-motor reaching task with false feedback [113]. It was also found that yoga practices can improve proprioceptive skills (i.e., higher accuracy of joint position) both in healthy individuals [114] and in congenitally blind young people [115].

In a qualitative study by Mehling and colleagues [8], the construct of BA and its rehabilitation in focus group practitioners was investigated. They found that the primary objective of mind–body approaches is to cultivate an embodied self, i.e., integration of various levels including body, mind, breath, emotion, and personality, aligning with the perspectives of phenomenological philosophers (such as Merleau-Ponty). The participants of this study confirmed the view that the self–body dialectic conceptualized by phenomenological philosophers needs to be extended in body–self–environment “trialectic”, because the person is embedded and active in a cultural environment and society [116]. Participants preferred the term “self-awareness” over body awareness, as the latter perpetuates the mind/body split. In summary, what has emerged from the focus groups, in fact, is that all participants commonly ground BA as an inseparable aspect of embodied self-awareness realized in action and in interaction with the world. Moreover, embodiment is viewed as an innate tendency towards emergent self-organization and wholeness, and through BA-enhancing therapies patients reported progress towards greater unity between body and self. These therapies facilitate a reconnection with the disrupted embodiment process and access the invisible integrity of the self. In order to understand the impact of body awareness-enhancing treatments and how they provide psychological and pain-related benefits, their findings suggest that it is necessary to broaden the biomedical paradigm to include a developmental model of embodiment, which overcomes the mind–body split.

### 7.2. Rehabilitation Approaches on Neurological Diseases

It has been shown that both central and peripheral stimulation, such as caloric vestibular stimulation (*CVS*) and transcranical direct current stimulation (*tDCS*), can have a temporary and selective positive impact on patients suffering from specific neurological disorders affecting some parts of BA, like unilateral spatial neglect, anosognosia, and somatoparaphrenia. For example, CVS seems to be efficient in restoring motor awareness [117,118], body ownership [119], and sensory perception [120,121]. In patients with SP, an increased in body temperature after CVS was observed, correlating with restored body ownership [122]. These effects suggest that physiological components play a significant role in conscious bodily experience and interact with cognitive processes. Also, tDCS has been effective in modulating motor awareness. For example, patients with AHP [123] showed improvements in motor awareness under tDCS over the right premotor cortex when they were asked to perform actions with their eyes open, but not in other conditions.

Other methods, like verbal [124] or spatial manipulations [119] and mirror techniques [125], have also shown promise in inducing selective remission in awareness disorders. Overall, these findings demonstrate the malleability of conscious processes and highlight the potential for stimulation techniques to selectively modulate awareness disorders.

The significance of BA resides in its ongoing monitoring, updating, and feedback provision concerning the positioning and movement of one’s body in space. It serves as the primary mechanism for integrating information for perception, decision-making, and action, underscoring the critical need for precise movement control based on accurate body information [126,127]. Recently, the role of BA in the recovery of stroke patients and their motor function has been investigated. Following a stroke, it has been observed that the majority of patients experience impairment in the sensation and perception of their own body, which influences their mental representation [128] and profoundly impacts their BA [129,130,131,132]. Altered processing in the motor and sensory cortices results in distorted body information, giving rise to diverse manifestations like the distorted perception of limb size, position, shape, or weight. Consequently, this hampers the accuracy and regulation of movements, including postural control, dynamic balance, and coordination, as well as the individual’s capacity to safely navigate their surroundings [127,129,130,131]. Furthermore, reduced BA seems to interfere with the rehabilitation process also, influencing its duration and discharge destination [129,131,133]. Previously, the beneficial impact of BA training on rehabilitative outcomes has already demonstrated, specifically in terms of balance and mobility [134,135,136]. Nevertheless, there is a lack of studies that have directly investigated and measured the effects of training directly on BA. An interesting study [67] explored the impairment of BA following a stroke and its recovery in the subsequent months. It was observed that BA was reduced after the stroke but tended to recover within the first month, a period associated with spontaneous upregulation of plasticity, similar to motor recovery process. Motor recovery, in fact, is most likely to occur within the initial 3–6 months, when neural plasticity is at its peak following a stroke. After this period, improvements may continue, level off, or even decline [137,138]. There appears to be a similar pattern between motor recovery and recovery of BA. Furthermore, it has been observed that BA is associated with clinical outcomes related to self-efficacy, quality of life, and motor function/impairment. Both questionnaires used to measure BA, however, showed weak correlations with perceived sensations (tactile and proprioceptive). Impairments in BA may therefore be partially explained by sensory loss and may be more dependent on other perceptual and conceptual processes.

### 7.3. Interoceptive Interventions and Psysiological Systems

Interoception deficits, which represent a “transdiagnostic” mechanism across multiple mental health disorders, are targeted by treatments affecting body physiology or cognitive processing of body signals. These interventions include neural stimulation (e.g., vagus nerve stimulation), pharmacological treatments (interoceptive immune or appetite pathway), and non-invasive interoception-based interventions (IBIs). IBIs focus on psychological and behavioral aspects. A recent systematic review by Heim and colleagues [25] of randomized control trials (RCTs) investigates, for the first time, the efficacy of IBIs at improving interoception and symptoms of mental disorders. They categorized IBIs into three groups:

(1) Perceptual bottom-up processing: physiological-focused interventions (e.g., breathing) without reflection or discussion.
(2) Metacognitive appraisal of reflections: reflection on bodily signals’ meaning without modifying them.
(3) Both: combining attention to bodily signals with reflection in therapy sessions. An example is “Basic body awareness therapy” (BBAT) which consists in movement and massage therapy, followed by reflections about the experience with the therapist.

Results from 31 RCTs showed IBIs significantly improved interoception in disorders like irritable bowel disease, fibromyalgia, PTSD, and substance use disorders. However, only 15/31 RCTs (48.4%) demonstrated significant improvement in mental health symptoms, notably for eating disorders, substance use disorders, and irritable bowel syndrome. IBIs of the third category (perceptual + appraisal) showed better symptom improvement compared to control conditions, suggesting targeting multiple interoceptive dimensions may be necessary in addition to IAcc enhancement. However, the efficacy of IBIs varies across disorders, and further research is needed to explore underlying mechanisms and specific subgroups’ responsiveness.

While neurostimulation and pharmacological treatments targeting interoception have shown promise [13,139,140,141,142,143], behavioral IBIs have not targeted specific interoceptive biomarkers for mental health, such as cardiac or gastrointestinal interoception. Thus, this review did not find evidence supporting the hypothesis that improving disturbed interoception reduces mental health symptoms, except for substance use disorders and irritable bowel syndrome.

In conclusion, while BA focuses on awareness and control of one’s body as a whole, interoception specifically targets the perception of internal bodily signals. Both concepts are important in neurological rehabilitation and can be integrated into treatment programs aimed at improving functionality and quality of life for patients with neurological conditions.

Most interoceptive interventions focus on the improvement of physiological systems such as neuromodulation of the vagus nerve, measures of cardiac interoceptive accuracy and sensibility, slow breathing to change respiratory rate and depth, and mindfulness-based interventions. However, low information is reported regarding other levels of manipulation of bodily system in rehabilitation and regarding neurological diseases.

It is evident that the scientific literature is heavily focused on the psychological aspect, while the literature on neurological disorders appears to be lacking. This gap in knowledge is concerning, and it is crucial that more attention is given to neurological disorders to better understand their complexities and develop effective treatments.

## 8. Discussion

In the present review, it has been revealed that body awareness is a complex and multidimensional construct and its relationships with interoception is far from being exhaustively explained.

In the text, it has been highlighted that self-report instruments for measuring body awareness specifically are limited, and the importance of validated, multidimensional approaches has been emphasized. Interoceptive sensibility, for example, evaluates individual differences in the perceived ability to detect internal body variations without saying if it is accurate. A possible strategy to reach this aim is to use both measures of interoceptive sensibility and measures of interoceptive accuracy to assess the association between subjective (perceived) and objective (actual) interoceptive ability.

Regarding interoception, Desmedt and colleagues [16], in their critical analysis of human interoception, highlighted important issues in the definition and conceptualization of its components, indicating two main discrepancies:

1. Differences between phenomenon-based and physiological-based definitions of the construct.
2. A lack of empirical convergence between measures supposed to assess its components.

Above were described homeostatic pathways involved in the processing of internal signals. Despite their involvement in CNS cardiac, respiratory, and gastrointestinal processing, the somatosensory (non-homeostatic) pathway also plays a role; in fact, internal states also involve non-homeostatic receptors, fibers, neurons, subcortical, and cortical regions. However, the physiological definition of interoception (activation of homeostatic pathways) contradicts the phenomenon-based definition, which refers to the processing of internal bodily states. This contradiction implies a lack conceptual clarity, which exposes the risk of low construct validity and replicability issues (like a mismatch between the construct and its measures). Adhering strictly to the prevailing physiological definition of interoception, which involves the processing of internal states regulated by homeostatic pathways, renders nearly any assessment of internal state processing in humans as impure and biased due to potential involvement of nonhomeostatic pathways.

The second issue is related with the most shared classifications (Garfinkel and Khalsa) that are conceptually useful to provide a common ground for communication between researchers, however conceptualization is an ongoing process. Indeed, empirical evidence suggests that the identified dimensions are largely detached from existing measures, as evidenced by the low convergence between measures underlying the same dimension and between different domains (e.g., [90,144]). This is a problem for the interpretation of current empirical results. Researchers may mistakenly assume that an effect should replicate another measure of interoception because the latter is believed to measure the same dimension. However, this measure may be largely uncorrelated with the original one, posing a major threat to the interpretation of current results.

An important issue is that actual BA and interoception definitions often focus on heterogeneous and wide dimensions, which lead to overgeneralization. For BA, often the terms consciousness and awareness are used in the same way, and some theoretical models still fail to report a clear definition about them at the moment (for a review about this argument, see Sattin et al. [5] and Seth and Bayne [145]). For interoception, at the same time, current conceptualizations have compelled researchers to conform and narrow their research inquiries due to their focus on specific abilities. In Garfinkel model, for example, the emphasis is on the detection abilities, neglecting other interoceptive phenomena like attention. Moreover, some questionnaires were developed not to measure the dimensions proposed with the current conceptualizations but to assess different goals; e.g., BAQ was developed to evaluate the tendency to pay attention to normal and non-emotive body processes [79], and the MAIA was created in anxiety research. The convergence validity of interoceptive assessment presents challenges for the interpretability and replicability of findings, indicating a need to refine conceptualizations or improve measures to better align with diverse dimensions.

In order to address these challenges, Desmedt et al. [16] have suggested the development of upcoming models that incorporate (1) constructs at various levels of specificity, thereby adopting a hierarchical structure (as outlined by Comrey [146] and Watson [147]) and (2) additional dimensions to encompass all interoceptive phenomena. They emphasized the importance of conducting future studies that incorporate various interoception measures, preferably in diverse conditions, and conducting factor or network analyses to delve into the underlying dimensions of the current reliable measures or validate previously established theoretical models. It is finally crucial to refrain from extrapolating conclusions based on specific measures to broader constructs or other bodily domains until there is substantial evidence of convergence among the measures.

Epistemologically speaking, the interaction that we experience with our own body is of special interest because when we interact with our body, we are both treating it as an internal object and as an external object at the same time [148]. This means that when I touch my own hand, that feeling is similar to what I feel when I touch an object of the outside world, but not quite the same because my own skin is able to signal to me that I am touching my hand and that my hand is being touched at the very same time. This very specific example has certainly something to do with the topic of myness or ownership and the question about the weight that our own body has in the definition of the self. The question at the basis of this larger issue is: is one the owner of one’s own body, or one is its own body? Regarding the explicative models described in this review, certainly, a physicalist approach [149] on such a matter would suggest that since the body is also the place of the brain, and the brain is the organ through which we are able to perceive, experience and interact, we must be our own body. On a more Cartesian level, on the other hand, one could consider the body as a very useful tool, but it remains just a physical tool separated from the mental. However, a Cartesian take on the body is arguably anachronistic, so much so that the theory of the bodily immunity to error was used to disprove Cartesian theories on body awareness [150,151]. This theory is a philosophical perspective developed by Gareth Evans in the field of philosophy of mind and knowledge. It argues that certain forms of knowledge, such as those based on sensory perception, are immune from errors, in the sense that they cannot be subject to doubt or mistake. Such a theory [151] has as its roots the idea that if a property is self-ascribed, such as the properties that we ascribe to ourselves when we are bodily aware, then such properties cannot be erroneous. The consequences of these approaches should be considered by the predictive coding methods described in this article, considering that a privileged access to one’s body both on an informational point of view and on a sensorial point of view could imply that the body and the self are not linked to each other, but they are each other because they retain both mental and physical properties.

On the other hand, a functionalist approach (functionalism in the philosophy of mind holds that a mental state is not determined by its internal structure, but rather by its function or role within a system) on bodily awareness would state that the body is actually the means through which one is able to perceive, experience, and interact. And, for this very reason perceptual systems are cognitive modules that are encapsulated in an informational dimension. Consequently, they are insensitive to our internal beliefs [152], therefore they can be described as systematically internal functions. For instance, the neurobiology of visual illusions shows that [153] even when an observer is made aware of the illusion, contextually dependent aspects of the situation in which the illusion is shown, such an illusion still seems illusory to the eyes of the beholder. This is coherent with a functionalist approach, which does not support a certain unity of the cognitive and the perceptual levels.

Finally, in a somewhat similar perspective, a multi-modal approach to BA [154] would support the facts that a sensory perspective is not enough to ascribe unity to our sensory reception because senses fail to give a complete account of bodily experiences; therefore, BA cannot be fully explained by a sensory-driven theory, but rather it is multimodal, i.e., it has to do with the entire biology of our bodies and with the entirety of our bodily functions also including hierarchic representation/structures. A pragmatical example of the implication of the above presented philosophical perspectives could be offered by phantom limb phenomena. Phantom sensation occurs more intensely on sites with a greater cortical representation (ankle, foot, toes, sole, and heel) but, as well, it changes during movement considering the difference in the distribution of body weight, for example, during the walking practice [155,156]. In the past, the close connection between structure and function was emphasized by Simmel [157] and Price [158], who in examining patients suffering from leprosy found that the lack of the phantom phenomenon may originate from the deficiency of the sensomotoric functions. Indeed, in those cases, the reduction of the function and morphology happened in a similar way. However, results on rehabilitation of the phantom limbs in amputee patients has revealed that body awareness is not influenced by the cortical representation of the different areas (in contrast to, for example, body schema representation, which could be influenced by wearing a prosthesis, while body awareness is not) [159]. BA seems to be a rather stable structure and does not change immediately after limb loss, but after a few months; although the amputee can see the prosthesis and sense the phantom limb, they do not consider it as their own since they are aware of its absence. It does not appear in the image sharpness because, probably, it could be a higher level of representation.

In conclusion, the need for an extensive viewpoint about the interaction of body representations and human actions/movements should be at the center of future research. We considered unavoidable the study of body representations in a pathological setting in which it is possible to study both behaviors after central nervous system lesions as well as after body lesions in a context of unaltered brain areas.

Our phenomenological experience of the body is shaped by early egocentric representations as well as an allocentric frame of reference, so future studies are needed in clinical settings in order to foster the way for a neuroscientific approach to rehabilitation developing protocols able to offer new solutions for the study and the re-habilitation of human movement.

## Figures and Tables

**Table 2:** Items constituting the BAQ [79] and the BPQ [76] as reported in the original article.

	The Body Awarenss Questionnaire (BAQ) by Shields, S.A., Mallory, M.E., and Simon, A. [34].	The Body Perceptions Questionnaire (BPQ) Short Form by Porges [31].
	Listed below are a number of statements regarding your sensitivity to normal, non-emotive body processes. For each statement, select a number from 1 to 7 that best describes how the statement describes you and place the number in the box to the right of the statement.	Please rate your awareness on each of the characteristics described below. Select the answer that most accurately describes you.
1	I notice differences in the way my body reacts to various foods.	Swallowing frequently
2	I can always tell when I bump myself whether or not it will became a bruise.	An urge to cough or clear my throat
3	I always know when I’ve expected myself to the point where I’ll be sore the next day.	My mouth being dry
4	I am always aware of changes in my energy level when I eat certain foods.	How fast I am breathing
5	I know in advance when I’m getting the flu.	Watering or tearing of my eyes
6	I know I’m running a fever without taking the temperature.	Noises associated with my digestion
7	I can distinguish between tiredness because of hunger and tiredness because of lack of sleep.	A swelling of my body or parts of my body
8	I can accurately predict what time of day lack of sleep will catch up with me.	An urge to defecate
9	I am aware of a cycle in my activity level throughout the day.	Muscle tension in my arms and legs
10	I don’t notice seasonal rhythms and cycles in the way my body functions.	A bloated feeling because of water retention
11	As soon as I wake up in the morning I know how much energy I’ll have during the day.	Muscle tension in my face
12	I can tell when I go bed how well I will sleep that night.	Goose bumps
13	I notice distinct body reactions when I am fatigued.	Stomach and gut pains
14	I notice specific body responses to changes in the weather.	Stomach distension or bloatedness
15	I can predict how much sleep I will need at night in order to wake up refreshed.	Palms sweating
16	When my exercise habits change, I can predict very accurately how that will affect my energy level.	Sweat on my forehead
17	There seems to be a “best” time for me to go to sleep at night.	Tremor in my lips
18	I notice specific bodily reactions to being overhungry.	Sweat in my armpits
19		The temperature of my face (especially my ears)
20		Grinding my teeth
21		General jitteriness
22		The hair on the back of my neck “standing up”
23		Difficulty in focusing
24		An urge to swallow
25		How hard my heart is beating
26		Feeling constipated
	Note. Item 10 is reverse scored. Each item is rated on a 7-point Likert scale ranging from not at all true about me (1) to very true about me (7).	Note. Each item is rated on a 5-point likert scale (never, occasionally, sometimes, usually, always)

**Table 3:** Assessment tools and therapeutic application for the different components of interoception.

	Interoceptive Accuracy (IAcc)	Interoceptive Sensibility (IS)	Interoceptive Awareness (IA)
Mode of Assessment	Objective behavioral tests or performance-based measures on various abilities related to diverse body systems. The more used refer to the cardiovascular system.	Subjective self-report questionnaires	Combination of objective and subjective measures. Measure of degree to which objective accuracy is associated with subjective confidence.
Assessment tools	Heartbeat detection/perception task or heartbeat counting task (HCT) [85] Heartbeat discrimination task (HDT) [86] Waterload test [87] Evaluate the bitterness level of a liquid [88] Reproduce limb position (proprioceptive acuity) [89] Balance on one leg with closed eyes [90] Discriminate the duration of respiratory occlusions [91]	BPQ [76] MAIA [9] Confidence rating using a visual analogue scale Five-Facet Mindfulness Questionnaire (FFMQ) Scale of body connection (SBC)	ROC curves mapping confidence–accuracy correlation (i.e., Pearson’s r)
Interoceptive trainings	Buddhist and contemplative practices like ACT and mindfulness treatment (e.g., MBCT) Training of interoceptive accuracy IBIs (e.g., Interoceptive exposure)		
Therapeutic applications	INTEROCEPTION Panic disorder Anxiety symptoms Depression Subclinical eating disorders Alexithymia Irritable bowel disease Substance use disorder Fibromyalgia PTSD	BODY AWARENESS Somatoparaphrenia (SP) Anosognosia for emiplegia (AHP) Anosognosia for hemianesthesia (AHA)	

Note: We have integrated the trainings and rehabilitation approaches because usually in the literature, a clear distinction is not made between the different components of interoception (for a detailed description of the assessment of the dimensions identified by Khalsa and colleagues, see [13,16]. They are not reported here because these exceed the scope of the current study).

## Data Availability

Not applicable.

## References

[1] Oliver G., Cameron M.D. (2001). Visceral Sensory Neuroscience: Interoception.

[2] Amianto F., Northoff G., Abbate Daga G., Fassino S., Tasca G.A. (2016). Is Anorexia Nervosa a Disorder of the Self? A Psychological Approach. Front. Psychol..

[3] Merleau-ponty M. (1945). Phénoménologie de La Perception (1945) Motivation.

[4] De Vignemont F. (2011). A Mosquito Bite against the Enactive Approach to Bodily Experiences. J. Philos..

[5] Sattin D., Magnani F.G., Bartesaghi L., Caputo M., Fittipaldo A.V., Cacciatore M., Picozzi M., Leonardi M. (2021). Theoretical Models of Consciousness: A Scoping Review. Brain Sci..

[6] Bennett David J., Hill C., Bennett D., Hill C. (2014). Sensory Integration and the Unity of Consciousness.

[7] Gurwitsch A. (1985). Marginal Consciousness.

[8] Mehling W.E., Wrubel J., Daubenmier J.J., Price C.J., Kerr C.E., Silow T., Gopisetty V., Stewart A.L. (2011). Body Awareness: A Phenomenological Inquiry into the Common Ground of Mind-Body Therapies. Philos. Ethics Humanit. Med..

[9] Mehling W.E., Price C., Daubenmier J.J., Acree M., Bartmess E., Stewart A. (2012). The Multidimensional Assessment of Interoceptive Awareness (MAIA). PLoS ONE.

[10] Ainley V., Tsakiris M. (2013). Body Conscious? Interoceptive Awareness, Measured by Heartbeat Perception, Is Negatively Correlated with Self-Objectification. PLoS ONE.

[11] Sherrington C.S. (1906). The Integrative Action of the Nervous System.

[12] Chen W.G., Schloesser D., Arensdorf A.M., Simmons J.M., Cui C., Valentino R., Gnadt J.W., Nielsen L., Hillaire-Clarke C., St Spruance V. (2021). The Emerging Science of Interoception: Sensing, Integrating, Interpreting, and Regulating Signals within the Self. Trends Neurosci..

[13] Khalsa S.S., Adolphs R., Cameron O.G., Critchley H.D., Davenport P.W., Feinstein J.S., Feusner J.D., Garfinkel S.N., Lane R.D., Mehling W.E. (2018). Interoception and Mental Health: A Roadmap. Biol. Psychiatry Cogn. Neurosci. Neuroimaging.

[14] Ceunen E., Vlaeyen J.W., Van Diest I. (2016). On the Origin of Interoception. Front. Psychol..

[15] Craig A.D. (2015). How Do You Feel? An Interoceptive Moment with Your Neurobiological Self.

[16] Desmedt O., Luminet O., Maurage P., Corneille O. (2023). Discrepancies in the Definition and Measurement of Human Interoception: A Comprehensive Discussion and Suggested Ways Forward. Perspect. Psychol. Sci..

[17] Critchley H.D., Harrison N.A. (2013). Visceral Influences on Brain and Behavior. Neuron.

[18] Paulus M.P., Tapert S.F., Schulteis G. (2009). The Role of Interoception and Alliesthesia in Addiction. Pharmacol. Biochem. Behav..

[19] Craig A.D. (2009). How Do You Feel–Now? The Anterior Insula and Human Awareness. Nat. Rev. Neurosci..

[20] Khalsa S.S., Lapidus R.C. (2016). Can Interoception Improve the Pragmatic Search for Biomarkers in Psychiatry?. Front. Psychiatry.

[21] Barrett L.F., Simmons W.K. (2015). Interoceptive Predictions in the Brain. Nat. Rev. Neurosci..

[22] Mehling W.E., Gopisetty V., Daubenmier J., Price C.J., Hecht F.M., Stewart A. (2009). Body Awareness: Construct and Self-Report Measures. PLoS ONE.

[23] Garfinkel S.N., Seth A.K., Barrett A.B., Suzuki K., Critchley H.D. (2015). Knowing Your Own Heart: Distinguishing Interoceptive Accuracy from Interoceptive Awareness. Biol. Psychol..

[24] Pollatos O., Herbert B.M., Hauke G., Kritikos A. (2018). Interoception: Definitions, Dimensions, Neural Substrates. Embodiment in Psychotherapy: A Practitioner’s Guide.

[25] Heim N., Bobou M., Tanzer M., Jenkinson P.M., Steinert C., Fotopoulou A. (2023). Psychological Interventions for Interoception in Mental Health Disorders: A Systematic Review of Randomized-Controlled Trials. Psychiatry Clin. Neurosci..

[26] Ainley V.A., Tajadura-Jiménez A., Fotopoulou M.T. (2012). Looking into Myself: The Effect of Self-Focused Attention on Interoceptive Sensitivity. Psychophysiology.

[27] Damasio A. (1999). The Feeling of What Happens: Body and Emotion in the Making of Consciousness.

[28] Dunn B.D., Galton H.C., Morgan R., Evans D., Oliver C., Meyer M., Cusack R., Lawrence A.D., Dalgleish T. (2010). Listening to Your Heart How Interoception Shapes Emotion Experience and Intuitive Decision Making. Psychol. Sci..

[29] Verdejo-Garcia A., Clark L., Dunn B.D. (2012). The Role of Interoception in Addiction: A Critical Review. Neurosci. Biobehav. Rev..

[30] Kenzie J.M., Semrau J.A., Findlater S.E., Yu A.Y., Desai J.A., Herter T.M., Hiil M.D., Scott S.H., Dukelow S.P. (2016). Localization of Impaired Kinesthetic Processing Post-Stroke. Front. Hum. Neurosci..

[31] Millidge B., Seth A., Buckley C.L. (2021). Predictive Coding: A Theoretical and Experimental Review. arXiv.

[32] Seth A.K., Friston K.J., Seth A.K. (2016). Active Interoceptive Inference and the Emotional Brain. Philos. Trans. R. Soc. B Biol. Sci..

[33] Ainley V., Apps M.A., Fotopoulou A., Tsakiris M. (2016). Bodily Precision: A Predictive Coding Account of Individual Differences in Interoceptive Accuracy. Philos. Trans. R. Soc. B Biol. Sci..

[34] Eshkevari E., Rieger E., Longo M.R., Haggard P., Treasure J. (2012). Increased Plasticity of the Bodily Self in Eating Disorders. Psychol. Med..

[35] Sattin D., Parma C., Lunetta C., Zulueta A., Lanzone J., Giani L., Vassallo M., Picozzi M., Parati E.A. (2023). An Overview of the Body Schema and Body Image: Theoretical Models, Methodological Settings and Pitfalls for Rehabilitation of Persons with Neurological Disorders. Brain Sci..

[36] Legrand N., Nikolova N., Correa C., Brændholt M., Stuckert A., Kildahl N., Vejlø M., Fardo F., Allen M. (2021). The Heart Rate Discrimination Task: A Psychophysical Method to Estimate the Accuracy and Precision of Interoceptive Beliefs. bioRxiv.

[37] Smith R., Kuplicki R., Feinstein J., Forthman K.L., Stewart J.L., Paulus M.P., Khalsa S.S. (2020). A Bayesian Computational Model Reveals a Failure to Adapt Intero- Ceptive Precision Estimates across Depression, Anxiety, Eating, and Substance Use Disorders. PLoS Comput. Biol..

[38] Friston K.A. (2005). Theory of Cortical Responses. Philos. Trans. R. Soc. B Biol. Sci..

[39] Fotopoulou A. (2014). Time to Get Rid of the ‘Modular’ in Neuropsychology: A Unified Theory of Anosognosia as Aberrant Predictive Coding. J. Neuropsychol..

[40] Fotopoulou A. (2015). The Virtual Bodily Self: Mentalisation of the Body as Revealed in Anosognosia for Hemiplegia. Conscious. Cogn..

[41] Apps M.A., Tsakiris M. (2014). The Free-Energy Self: A Predictive Coding Account of Self-Recognition. Neurosci. Biobehav. Rev..

[42] Hopp W. (2011). Perception and Knowledge: A Phenomenological Account.

[43] Clark A. (2017). Busting out: Predictive Brains, Embodied Minds, and the Puzzle of the Evidentiary Veil. Noûs.

[44] Zahavi D. (2018). Brain, Mind, World: Predictive Coding, Neo-Kantianism, and Transcendental Idealism. Husserl. Stud..

[45] Krause T., Spindler L., Poeck B., Strauss R. (2019). Drosophila Acquires a Long-Lasting Body-Size Memory from Visual Feedback. Curr. Biol..

[46] Tanimoto H. (2019). Bodily Awareness: How Flies Learn Their Own Body Size. Curr. Biol..

[47] Middlebrooks E.H., Grewal S.S. (2022). Brain Connectomics. Neuroimaging Clin. N. Am..

[48] Brugger P., Kollias S.S., Müri R.M., Crelier G., Hepp-Reymond M.C., Regard M. (2000). Beyond Re-Membering: Phantom Sensations of Congenitally Absent Limbs. Proc. Natl. Acad. Sci. USA.

[49] Gogolla N. (2017). The Insular Cortex. Curr. Biol..

[50] Critchley H.D., Wiens S., Rotshtein P., Ohman A., Dolan R.J. (2004). Neural Systems Supporting Interoceptive Awareness. Nat. Neurosci..

[51] Critchley H.D., Garfinkel S.N. (2017). Interoception and Emotion. Curr. Opin. Psychol..

[52] Singer T., Critchley H.D., Preuschoff K. (2009). A Common Role of Insula in Feelings, Empathy and Uncertainty. Trends Cogn. Sci..

[53] Paulus M.P., Feinstein J.S., Khalsa S.S. (2019). An Active Inference Approach to Interoceptive Psychopathology. Annu. Rev. Clin. Psychol..

[54] Kusumoto-Yoshida I., Liu H., Chen B.T., Fontanini A., Bonci A. (2015). Central Role for the Insular Cortex in Mediating Conditioned Responses to Anticipatory Cues. Proc. Natl. Acad. Sci. USA.

[55] Naqvi N.H., Bechara A. (2009). The Hidden Island of Addiction: The Insula. Trends Neurosci..

[56] Ibañez A., Gleichgerrcht E., Manes F. (2010). Clinical Effects of Insular Damage in Humans. Brain Struct. Funct..

[57] Babinski J. (1914). Contribution to the Study of Mental Disorders in Organic Cerebral Hemiplegia (Anosognosia). Rev. Neurol..

[58] Moro V., Pernigo S., Zapparoli P., Cordioli Z., Aglioti S.M. (2011). Phenomenology and Neural Correlates of Implicit and Emergent Motor Awareness in Patients with Anosognosia for Hemiplegia. Behav. Brain Res..

[59] Marcel A.J., Tegnér R., Nimmo-Smith I. (2004). Anosognosia for Plegia: Specificity, Extension, Partiality and Disunity of Bodily Unawareness. Cortex.

[60] Berti A., Bottini G., Gandola M., Pia L., Smania N., Stracciari A., Castiglioni I., Vallar G., Paulesu E. (2005). Shared Cortical Anatomy for Motor Awareness and Motor Control. Science.

[61] Karnath H.O., Baier B., Nägele T. (2005). Awareness of the Functioning of One’s Own Limbs Mediated by the Insular Cortex?. J. Neurosci..

[62] Fotopoulou A., Pernigo S., Maeda R., Rudd A., Kopelman M.A. (2010). Implicit Awareness in Anosognosia for Hemiplegia: Unconscious Interference without Conscious Re-Representation. Brain.

[63] Moro V., Pernigo S., Tsakiris M., Avesani R., Edelstyn N.M.J., Jenkinson P.M., Fotopoulou A. (2016). Motor versus Body Awareness: Voxel-Based Lesion Analysis in Anosognosia for Hemiplegia and Somatoparaphrenia Following Right Hemisphere Stroke. Cortex.

[64] Bottini G., Sedda A., Ferre E.R., Invernizzi P., Gandola M., Paulesu E. (2009). Productive Symptoms in Right Brain Damage. Curr. Opin. Neurol..

[65] Romano D., Gandola M., Bottini G., Maravita A. (2014). Somatoparaphrenia and Anosognosia: Clues to Body Awareness. Brain.

[66] de Vignemont F. (2010). Body Schema and Body Image-Pros and Cons. Neuropsychologia.

[67] Serrada I., Hordacre B., Hillier S. (2021). Recovery of Body Awareness After Stroke: An Observational Study. Front. Neurol..

[68] Moro V., Besharati S., Scandola M., Bertagnoli S., Ponzo S., Bulgarelli C., Fotopoulou A., Paul M., Moro V., Besharati S. (2021). The Motor Unawareness Assessment ( MUNA ): A New Tool for the Assessment of Anosognosia for Hemiplegia The Motor Unawareness Assessment (MUNA): A New Tool for the Assessment of ABSTRACT. J. Clin. Exp. Neuropsychol..

[69] Anderson R. (2006). Body Intelligence Scale: Defining and Measuring the Intelligence of the Body. Humanist. Psychol..

[70] Daubenmier J. (2005). The Relationship of Yoga, Body Awareness, and Body Responsiveness to Self-Objectification and Disordered Eating. Psychol. Women Q..

[71] Forester C.A. (2001). Body Awareness: An Aspect of Countertransference Management that Moderates Vicarious Traumatization.

[72] Franzoi S.L., Kessenich J.J., Sugrue P.A. (1989). Gender Differences in the Experience of Body Awareness: An Experiential Sampling Study. Sex Roles.

[73] Hansell S., Sherman G., Mechanic D. (1991). Body Awareness and Medical Care Utilization among Older Adults in an HMO. J. Gerontol. Soc. Sci..

[74] Lombardo C., San Martini P., Violani C. (1995). The Factorial Components and Psychometric Characteristics of a Questionnaire on Body Awareness/Composizione Fattoriale e Caratteristiche Psicometriche Di Un Questionario Di Consapevolezza Corporea (QCC). Boll. Psicol. Appl..

[75] Miller L.C., Murphy R., Buss A.H. (1981). Consciousness of Body: Private and Public. J. Personal. Soc. Psychol..

[76] Porges S.W. (1993). Body Perception Questionnaire. https://www.traumascience.org/body-perception-questionnaire.

[77] Price C.J., Thompson E.A. (2007). Measuring Dimensions of Body Connection: Body Awareness and Bodily Dissociation. J. Altern. Complement. Med..

[78] Schmidt N.B., Lerew D.R., Trakowski J.H. (1997). Body Vigilance in Panic Disorder: Evaluating Attention to Bodily Perturbations. J. Consult. Clin. Psychol..

[79] Shields S.A., Mallory M.E., Simon A. (1989). The Body Awareness Questionnaire: Reliability and Validity. J. Pers. Assess..

[80] Snell W.J., Johnson G. (1997). The Multidimensional Health Questionnaire. Am. J. Health Behav..

[81] Wiens S. (2005). Interoception in Emotional Experience. Curr. Opin. Neurol..

[82] Fairclough S.H., Goodwin L. (2007). The Effect of Psychological Stress and Relaxation on Interoceptive Accuracy: Implications for Symptom Perception. J. Psychosom. Res..

[83] Pauels M. (2017). L’Effet d’un Programme d’Entraînement à La Pleine Conscience Sur La Perception Des Sensations Corporelles Emotion-Nelles: Une Etude Contrôlée.

[84] Lewis J.S. (2010). Body Perception Disturbance (BPD) in CRPS. Pract. Pain Manag..

[85] Schandry R. (1981). Heart Beat Perception and Emotional Experience. Psychophysiology.

[86] Whitehead W.E., Drescher V.M., Heiman P., Blackwell B. (1977). Relation of Heart Rate Control to Heartbeat Per-Ception. Biofeedback Self. Regul..

[87] Van Dyck Z., Vögele C., Blechert J., Lutz A.P., Schulz A., Herbert B.M. (2016). The Water Load Test as a Measure of Gastric Interoception: Development of a Two-Stage Protocol and Application to a Healthy Female Population. PLoS ONE.

[88] Ferentzi E., Köteles F., Csala B., Drew R., Tihanyi B.T., Pulay-Kottlár G., Doering B.K. (2017). What Makes Sense in Our Body? Personality and Sensory Correlates of Body Awareness and Somatosensory Amplification. Personal. Individ. Differ..

[89] Goble D.J. (2010). Proprioceptive Acuity Assessment via Joint Position Matching: From Basic Science to General Practice. Phys. Ther..

[90] Ferentzi E., Bogdány T., Szabolcs Z., Csala B., Horváth Á., Köteles F. (2018). Multichannel Investigation of Interoception: Sensitivity Is Not a Generalizable Feature. Front. Hum. Neurosci..

[91] Van Den Houte M., Vlemincx E., Franssen M., Diest I.V., Oudenhove L.V., Luminet O. (2021). The Respiratory Occlusion Discrimination Task: A New Paradigm to Measure Respiratory Interoceptive Accuracy. Psychophysiology.

[92] Sherman K.J., Cherkin D.C., Erro J., Miglioretti D.L., Deyo R.A. (2005). Comparing Yoga, Exercise, and a Self-Care Book for Chronic Low Back Pain: A Randomized, Controlled Trial. Ann. Intern. Med..

[93] Smith M.C., Stallings M.A., Mariner S., Burrall M. (1999). Benefits of Massage Therapy for Hospitalized Patients: A Descriptive and Qualitative Evaluation. Altern. Ther. Health Med..

[94] Madore A., Kahn J., Audette J.F., Bailey A. (2008). Therapeutic Massage and Bodywork in integrative pain management. Integrative Pain Medicine.

[95] Price C. (2005). Body-Oriented Therapy in Recovery from Child Sexual Abuse: An Efficacy Study. Altern. Ther. Health Med..

[96] Lazar S.W., Kerr C.E., Wasserman R.H., Gray J.R., Greve D.N., Treadway M.T., McGarvey M., Quinn B.T., Dusek J.A., Benson H. (2005). Meditation Experience Is Associated with Increased Cortical Thickness. Neuroreport.

[97] Ives J.C. (2003). Comments on “the Feldenkrais Method: A Dynamic Approach to Changing Motor Behavior”. Res. Q. Exerc. Sport.

[98] Ernst E., Canter P.H. (2003). The Alexander Technique: A Systematic Review of Controlled Clinical Trials. Forsch. Komplementärmedizin Klass. Naturheilkunde.

[99] Mehling W.E. (2001). The Experience of Breath as a Therapeutic Intervention—Psychosomatic Forms of Breath Therapy. A Descriptive Study about the Actual Situation of Breath Therapy in Germany, Its Relation to Medicine, and Its Application in Patients with Back Pain. Forsch. Komplementärmedizin Klass. Naturheilkunde.

[100] Ryding C., Rudebeck E.C., Roxendal G. (2002). Assessing Body Awareness in Healthy Subjects—The First Steps toward the Construction of the BAS-Health. Adv. Physiother..

[101] Landsman-Dijkstra J.J., van Wijck R., Groothoff J.W. (2006). The Long-Term Lasting Effectiveness on Self-Efficacy, Attribution Style, Expression of Emotions and Quality of Life of a Body Awareness Program for Chronic a-Specific Psychosomatic Symptoms. Patient Educ. Couns..

[102] Bishop S.R., Lau M., Shapiro S., Carlson L., Anderson N.D., Carmody J., Segal Z.V., Abbey S., Speca M., Velting D. (2004). Mindfulness: A Proposed Operational Definition. Clin. Psychol. Sci. Pract..

[103] Pérez-Peña M., Notermans J., Desmedt O., Van der Gucht K., Philippot P. (2022). Mindfulness-Based Interventions and Body Awareness. Brain Sci..

[104] Williams J.M.G., Broadbent K. (1987). Autobiographical Memory in Suicide Attempters. J. Abnorm. Psychol..

[105] Gu J., Strauss C., Bond R., Cavanagh K. (2015). How Do Mindfulness-Based Cognitive Therapy and Mindfulness-Based Stress Reduction Improve Mental Health and Wellbeing ? A Systematic Review and Meta-Analysis of Mediation Studies. Clin. Psychol. Rev..

[106] Luberto C.M., Cotton S., McLeish A.C., Mingione C.J., O’Bryan E.M. (2015). Mindfulness Skills and Emotion Regulation: TheMediating Role of Coping Self-Efficacy. Mindfulness.

[107] Fissler M., Winnebeck E., Schroeter T., Gummersbach M., Huntenburg J.M., Gaertner M., Barnhofer T. (2016). An Investigation of the Effects of Brief Mindfulness Training on Self-Reported Interoceptive Awareness, the Ability to Decenter, and Their Role in the Reduction of Depressive Symptoms. Mindfulness.

[108] Forkmann T., Scherer A., Meessen J., Michal M., Schächinger H., Vögele C., Schulz A. (2016). Making Sense of What You Sense: Disentangling Interoceptive Awareness, Sensibility and Accuracy. Int. J. Psychophysiol..

[109] Desmedt O., Luminet O., Corneille O. (2018). The Heartbeat Counting Task Largely Involves Non-Interoceptive Processes: Evidence from Both the Original and an Adapted Counting Task. Biol. Psychol..

[110] Kabat-Zinn J. (2013). Full Catastrophe Living: How to Cope with Stress, Pain and Illness Using Mindfulness Meditation.

[111] Segal Z.V., Williams M., Teasdale J. (2018). Mindfulness-Based Cognitive Therapy for Depression.

[112] De Jong M., Lazar S.W., Hug K., Mehling W.E., Hölzel B.K., Sack A.T., Peeters F., Ashih H., Mischoulon D., Gard T. (2016). Effects of Mindfulness-Based Cognitive Therapy on Body Awareness in Patients with Chronic Pain and Comorbid Depression. Front. Psychol..

[113] Naranjo J.R., Schmidt S. (2012). Is It Me or Not Me? Modulation of Perceptual-Motor Awareness and Visuomotor Performance by Mindfulness Meditation. BMC Neurosci..

[114] Telles S., Naveen K.V., Shreevidya N. (2007). A Comparison of the Bilateral Elbow Joint Position Sense in Yoga and Non Yoga Practitioners. J. Indian Psychol..

[115] Mohanty S., Pradhan B., Nagathna R. (2014). The Effect of Yoga Practice on Proprioception in Congenitally Blind Students. Br. J. Vis. Impair..

[116] Hudak P.L., McKeever P., Wright J.G. (2007). Unstable Embodiments: A Phenomenological Interpretation of Patient Satisfaction with Treatment Outcome. J. Med. Humanit..

[117] Vallar G., Sterzi R., Bottini G., Cappa S., Rusconi M.L. (1990). Temporary Remission of Left Hemianesthesia after Vestibular Stimulation. A Sensory Neglect Phenomenon. Cortex.

[118] Vallar G., Bottini G., and Sterzi R. (2003). Anosognosia for Left-Sided Motor and Sensory Deficits, Motor Neglect, and Sensory Hemiinattention: Is There a Relationship?. Prog. Brain Res..

[119] Salvato G., Gandola M., Veronelli L., Agostoni E.C., Sberna M., Corbo M., Bottini G. (2016). The Spatial Side of Somatoparaphrenia: A Case Study. Neurocase.

[120] Bottini G., Paulesu E., Sterzi R., Warburton E., Wise R.J., Vallar G., Frackowiak R.S.J., Frith C.D. (1995). Modulation of Conscious Experience by Peripheral Sensory Stimuli. Nature.

[121] Bottini G., Paulesu E., Gandola M., Loffredo S., Scarpa P., Sterzi R., Santilli I., Defanti C.A., Scialfa G., Fazio F. (2005). Left Caloric Vestibular Stimulation Ameliorates Right Hemianesthesia. Neurology.

[122] Salvato G., Gandola M., Veronelli L., Berlingeri M., Corbo M., Bottini G. (2018). The Vestibular System, Body Temperature and Sense Ofbody Ownership: A Potential Link? Insights from a Single Case Study. Physiol. Behav..

[123] Gandola M., Sedda A., Manera M., Pingue V., Salvato G., Spitoni G.F., Pistarini C., Giorgi I., Pizzamiglio L., Bottin G.i. (2014). Selective Improvement of Anosognosia for Hemiplegia during Transcranial Direct Current Stimulation: A Case Report. Cortex.

[124] Bottini G., Bisiach E., Sterzi R., Vallar G. (2002). Feeling Touches in Someone Else’s Hand. Neuroreport.

[125] Jenkinson P.M., Haggard P., Ferreira N.C., Fotopoulou A. (2013). Body Ownership and Attention in the Mirror: Insights from Somatoparaphrenia and the Rubber Hand Illusion. Neuropsychologia.

[126] Williams L. (2014). Investigating the Disturbance of Body Schema in Stroke.

[127] Ahn S.N. (2018). Differences in Body Awareness and Its Effects on Balance Function and Independence in Activities of Daily Living for Stroke. J. Phys. Ther. Sci..

[128] Stott H., Cramp M., McClean S., Turton A. (2021). ‘Somebody Stuck Me in a Bag of Sand’: Lived Experiences of the Altered and Uncomfortable Body after Stroke. Clin. Rehabil..

[129] Carey L.M., Macdonell R., Matyas T. (2011). SENSe: Study of the Effectiveness of Neurorehabilitation on Sensation: A Randomized Controlled Trial. Neurorehabil. Neural Repair.

[130] Carey L.M., Matyas T. (2011). Frequency of Discriminative Sensory Loss in the Hand after Stroke in a Rehabilitation Setting. J. Rehabil. Med..

[131] Carey L.M., Matyas T.A., Baum C. (2018). Effects of Somatosensory Impairment on Participation after Stroke. Am. J. Occup. Ther..

[132] Sullivan J.E., Hedman L. (2008). Sensory Dysfunction Following Stroke: Incidence, Significance, Examination, and Intervention. Top. Stroke Rehabil..

[133] Carey L.M., Jacobs S., Baum C., Connor L. Loss of Somatosensation and Its Impact on Activity Participation Following Stroke. Proceedings of the 2010 European Stroke Conference.

[134] Bang D.H., Cho H. (2016). Effect of Body Awareness Training on Balance and Walking Ability in Chronic Stroke Patients: A Randomized Controlled Trial. J. Phys. Ther. Sci..

[135] Batson G., Deutsch J. (2005). Effects of Feldekrais Awareness through Movement on Balance in Adults with Chronic Neurological Deficits Following Stroke: A Preliminary Study. Complement. Health Pract. Rev..

[136] Lindvall M.A., Forsberg A. (2014). Body Awareness Therapy in Persons with Stroke: A Pilot Randomized Controlled Trial. Clin. Rehabil..

[137] Langhorne P., Bernhardt J., Kwakkel G. (2011). Stroke Rehabilitation. Lancet.

[138] Murphy T.H., Corbett D. (2009). Plasticity during Stroke Recovery: From Synapse to Behaviour. Nat. Rev. Neurosci..

[139] Nord C.L., Garfinkel S.N. (2022). Interoceptive Pathways to Understand and Treat Mental Health Conditions. Trends Cogn. Sci..

[140] Weng H.Y., Feldman J.L., Leggio L., Napadow V., Park J., Price C.J. (2021). Interventions and Manipulations of Interoception. Trends Neurosci..

[141] Schoeller F., Haar A.J.H., Jain A., Maes P. (2019). Enhancing Human Emotions with Interoceptive Technologies. Phys. Life Rev..

[142] Livermore J.J., Holmes C.L., Moga G., Adamatzky K., Critchley H.D., Garfinkel S.N., Campbell-Meiklejohn D. (2022). A Single Oral Dose of Citalopram Increases Interoceptive Insight in Healthy Volunteers. Psycho-Pharmacology.

[143] Paulus M.P., Stewart J.L., Haase L. (2013). Treatment Approaches for Interoceptive Dysfunctions in Drug Addiction. Front. Psychiatry.

[144] Desmedt O., Heeren A., Corneille O., Luminet O. (2022). What Do Measures of Self-Report Interoception Measure? Insights from a Systematic Review, Latent Factor Analysis, and Network Approach. Biol. Psychol..

[145] Seth A.K., Bayne T. (2022). Theories of Consciousness. Nat. Rev. Neurosci..

[146] Comrey A.L. (1988). Factor-Analytic Methods of Scale Development in Personality and Clinical Psychology. J. Consult. Clin. Psychol..

[147] Watson D., Clark L.A., Harkness A.R. (1994). Structures of Personality and Their Relevance to Psychopathology. J. Abnorm. Psychol..

[148] Morton A. (1980). The Will: A Dual Aspect Theory. Philos. Rev..

[149] Churchland M.P. (1996). The Engine of Reason, The Seat of the Soul: A Philosophical Journey into the Brain.

[150] Cassam Q., Bermudez J.L., Anthony J.M., Eilan N.M. (1995). Introspection and Bodily Self–Ascription. The Body and The Self.

[151] Evans G., McDowell J. (1982). The Varieties of Reference.

[152] Fodor J. (1985). The Modularity of Mind. Philos. Rev..

[153] Eagleman D.M., Sejnowski T.J. (2001). The Flash-Lag Illusion: Distinguishing a Spatial from a Temporal Effect, and Why That Matters for Interpreting Visual Physiology. J. Vis..

[154] De Vignemont F. (2014). A Multimodal Conception of Bodily Awareness. Mind.

[155] Davis R. (1993). Phantom Sensation, Phantom Pain, and Stump Pain. Arch. Phys. Med. Rehabil.

[156] Giordano A., Boffano M., Piana R., Mutani R., Cicolin A. (2021). Body Schema Self-Awareness and Related Dream Content Modifications in Amputees Due to Cancer. Brain Sci..

[157] Simmel M.L. (1956). Phantoms in Patients with Leprosy and in Elderly Digital Amputees. Am. J. Psychol..

[158] Price D.B. (1976). Phantom Limb Phenomenon in Patients with Leprosy. J. Nerv. Ment. Dis..

[159] Mayer A., Kudar K., Bretz K., Tihanyi J. (2008). Body Schema and Body Awareness of Amputees. Prosthet. Orthot. Int..

